# The electron as a probe to measure the thickness distributions of electroactive films†
†Electronic supplementary information (ESI) available. See DOI: 10.1039/c9sc03653a


**DOI:** 10.1039/c9sc03653a

**Published:** 2019-11-18

**Authors:** Darren Buesen, Huaiguang Li, Nicolas Plumeré

**Affiliations:** a Center for Electrochemical Sciences – CES , Ruhr-Universität Bochum , 44780 Bochum , Germany . Email: nicolas.plumere@rub.de

## Abstract

Electron conducting films are ubiquitous in applications such as energy conversion, and their ability to fulfill their catalytic function can be greatly limited by inhomogeneities in their thickness or breaks within the film. Knowing the electroactive film thickness distribution would greatly facilitate optimization efforts, but techniques to measure this are lacking. Here, we present an electroanalytical method that provides the thickness distribution of the electrochemically accessible fraction of redox-active films in which the transfer of electrons is diffusional, *i.e.* by electron hopping. In this method, as the time scale of the experiment (the scan rate) is changed, the location of the diffusion layer boundary relative to the film roughness features is varied, allowing for the extraction of the film thickness distribution. In addition to being conveniently carried out in the solvated state, which is often the operational state of these conductive films, this approach is highly complementary to classical microscopy methods since it samples the entire modified electrode and is specific to the electroactive portions of the film. Therefore, this approach provides information on film morphology that is truly relevant for the catalytic processes being optimized, and thus can guide the optimization of catalyst integration in films towards macroscale cohesion and thickness homogeneity which are essential for optimal performances.

## Abstract 2

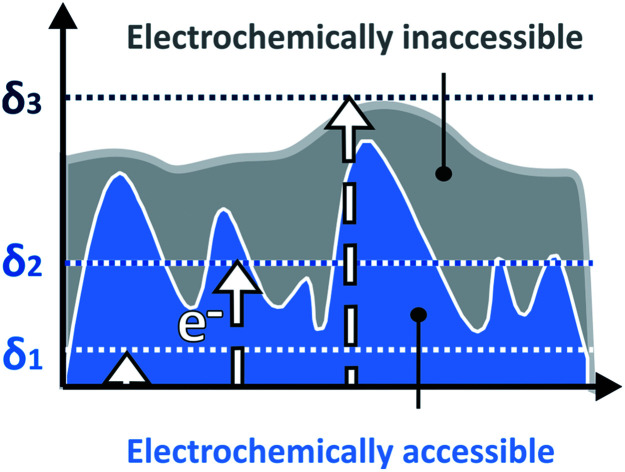
A theoretical model combined to an experimental study shows that the morphology of electron conducting films can be quantified directly from the analysis of cyclic voltammetry data.

## Introduction

Redox-active or conductive films assembled from synthetic^1^,^2^ or natural materials^3^ are ubiquitous as matrices for the immobilization and electrical wiring of catalysts or of light absorbing materials for technological applications. The most prominent of these are thin films based on perovskites and organic semiconductors^4^ for photovoltaics,^5^ as well as inorganic catalysts for reactions such as proton reduction and water oxidation.^6^,^7^ In addition, redox films containing biological or molecular catalysts immobilized on electrode surfaces have found numerous applications in sensing,^1^ and recently, have also attracted interest in energy conversion^8^–^12^ as well as in the electrosynthesis of small molecules.^13^

While design and optimization is often focused on the active components embedded within the film, the geometry and dimensions of the redox-active matrix also play a central role in the resultant catalytic or photoactive properties.^14^–^17^ For example, film thickness can be modulated to maximize the overall performance with respect to electron transfer, mass transport and catalyst loading and thus exploit catalytic systems to their full potential. Recently, the properties and thickness of a redox-active film were engineered to provide protection to sensitive catalysts,^10^,^12^,^18^ and may eventually serve to control reaction selectivity^19^ by regulating the local supply of electrons and reactants. Theoretical models describing reaction/diffusion processes in these systems,^6^,^15^–^17^ as well as mechanisms for protection^12^,^14^ with film thickness as a major design parameter, are in place to enable the rational optimization of their catalytic properties.

The central role of film thickness also means that the homogeneity of its distribution will likewise have a major impact on catalytic performance. For instance, a film with a highly heterogeneous thickness is detrimental because it may include areas where mass transport limits the catalytic performance due to excessive thickness, while on other areas of the same electrode, the catalyst loading may be limiting due to insufficient thickness. Therefore, for the optimization of such films, the homogeneity of the electroactive film thickness must be known.

A variety of confocal or atomic force microscopy (AFM) methods exist to characterize film morphology.^20^ However, these methods are often too complex for routine implementation or deliver only partial information. For instance, AFM is often applied, but only yields the top roughness of the sampled fraction of the film. Estimation of the thickness distribution is possible through profile measurements, but this requires partial film removal as an additional destructive step. Moreover, essential information, such as the thickness distribution of the electroactive fraction of the film, which is most relevant for electrocatalytic or light-induced charge transfer processes, remains inaccessible through AFM or confocal microscopy investigations.

Here, we propose a straightforward and non-destructive electroanalytical method, based on linear sweep voltammetry, that delivers the electroactive film thickness distribution directly, and under the conditions relevant for the catalytic processes being optimized. Since the films under consideration for these applications are intrinsically electron conducting, we can use the electron as a probe for quantifying the locations of the film boundaries with respect to the electrode.

**Fig. 1:**
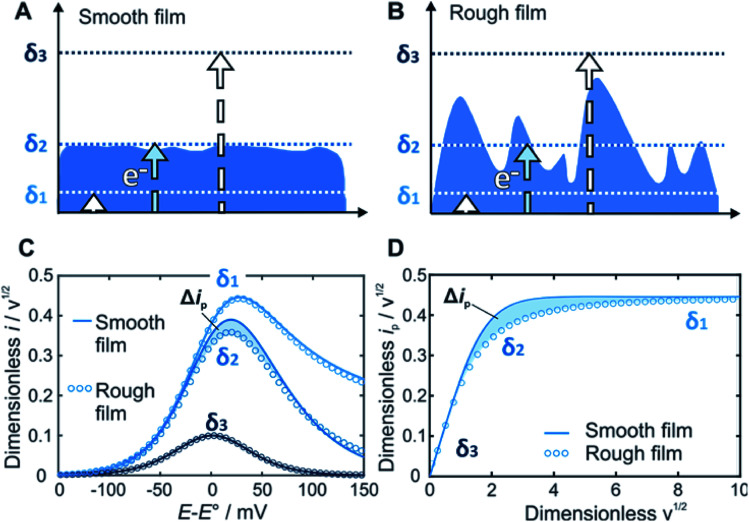
Modulating the diffusion layer thicknesses for deducing the film thickness distributions by linear sweep voltammetry (LSV). Schematic illustration of the diffusion layers of the electron (*δ*, dotted lines) defined by the time scale of the experiment as a function of the film boundary for (A) a smooth film, and for (B) a rough film. At the fastest scan rates, the corresponding diffusion layer is confined within the film boundary (*δ*_1_). At intermediate scan rates, the diffusion layer passes through the roughness features of the film (*δ*_2_). At the slowest scan rates, the diffusion layer goes beyond the outermost film boundary (*δ*_3_). (C) Corresponding LSVs (*w*_avg_^1/2^ = 10, 2 and 0.4) and (D) normalized peak current (*i*_p_) plot, for a smooth film (solid line, shape factor = 100), and for a rough film (blue circles, shape factor = 2). The difference in current responses (Δ*i*_p_, shaded blue areas) at intermediate diffusion layer thicknesses, allows for determination of the underlying film thickness distribution.

Within redox-active films, electrons are transported by a hopping mechanism between the tethered redox moieties at a rate which is defined by the apparent diffusion coefficient of the electron^21^ (*D*). For a given time scale (*θ*) defined by the scan rate (*ν*), the electron will travel a distance determined by the diffusion layer thickness (*δ*) defined as *δ* = (*Dθ*)^1/2^ = (*DRT*/*nFν*)^1/2^. The key feature of our method is that the current response related to the electron transfer within the film depends on the relative dimension of *δ* with respect to the film thickness *d* (Fig. 1). In contrast to a smooth film (Fig. 1A), the time scale window corresponding to *δ* values that reaches the outermost film boundaries is larger for rough films (Fig. 1B). Accordingly, the current response for a rough film deviates from the one obtained for a smooth film (Fig. 1C and D).

We exploit these deviations in peak currents obtained from linear sweep voltammograms to directly quantify the film thickness distribution. We demonstrate that the arrangement of the surface features does not significantly impact the accuracy of the thickness distribution determination when counter-ion transport is non-limiting. Major advantages of using this electrochemical method include (i) its simplicity, since only voltammetric measurements are required, (ii) its scope, since the thickness distribution is obtained for the entire film in contrast to a limited sampled area, and (iii) its relevance to the intended electrocatalytic application, since it specifically probes the electroactive fraction of the film.

## The model

### Linear sweep voltammetry with normalized parameters and a characteristic plot

For the case of planar diffusion in a perfectly smooth film,^22^ the theoretical position of the diffusion layer thickness (*δ*) with respect to the film thickness (*d*) can be expressed in terms of a dimensionless parameter (*w*^1/2^) according to eqn (1). Since *w*^1/2^ is proportional to *ν*^1/2^, *w*^1/2^ can be regarded as a normalization of the square root of the scan rate.1
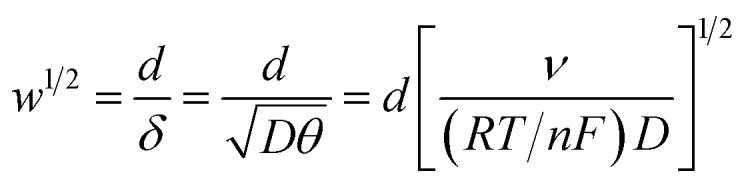

*R* is the gas constant, *T* is the temperature, *F* is the Faraday constant and *n* is the number of electrons exchanged. The peak current (*i*_p_) obtained at a given scan rate is normalized according to eqn (2), and can be plotted against the dimensionless parameter (*w*^1/2^), given by eqn (1), to prepare the characteristic plot for this system.2
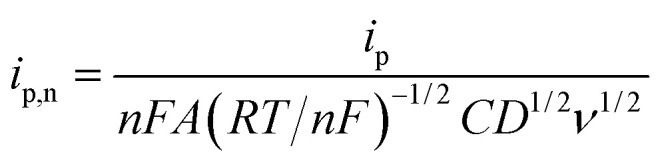

*A* is the surface area of the electrode and *C* is the concentration of the redox-active species within the film. Although there are several variables in these expressions, a plot of *i*_p,n_*vs. w*^1/2^ can be regarded as essentially the normalization of an *i*_p_/*ν*^1/2^*vs. ν*^1/2^ plot (Fig. 1D).

### Weibull distribution for the parameterization of film thickness variations

We use the Weibull distribution^23^ for the parameterization of film thickness distributions due to its ability to characterize films with extremely high inhomogeneity. The Weibull distribution is usually given as a shape factor (SF) which directly correlates with the relative standard deviation of the film thickness (see details in ESI Section S1†). Low values of the shape factor correspond to high relative standard deviations. Therefore, low shape factors correspond to rough films, and high shape factors correspond to smooth films.

### The deconstruction method for a planar electron diffusion reference

**Fig. 2:**
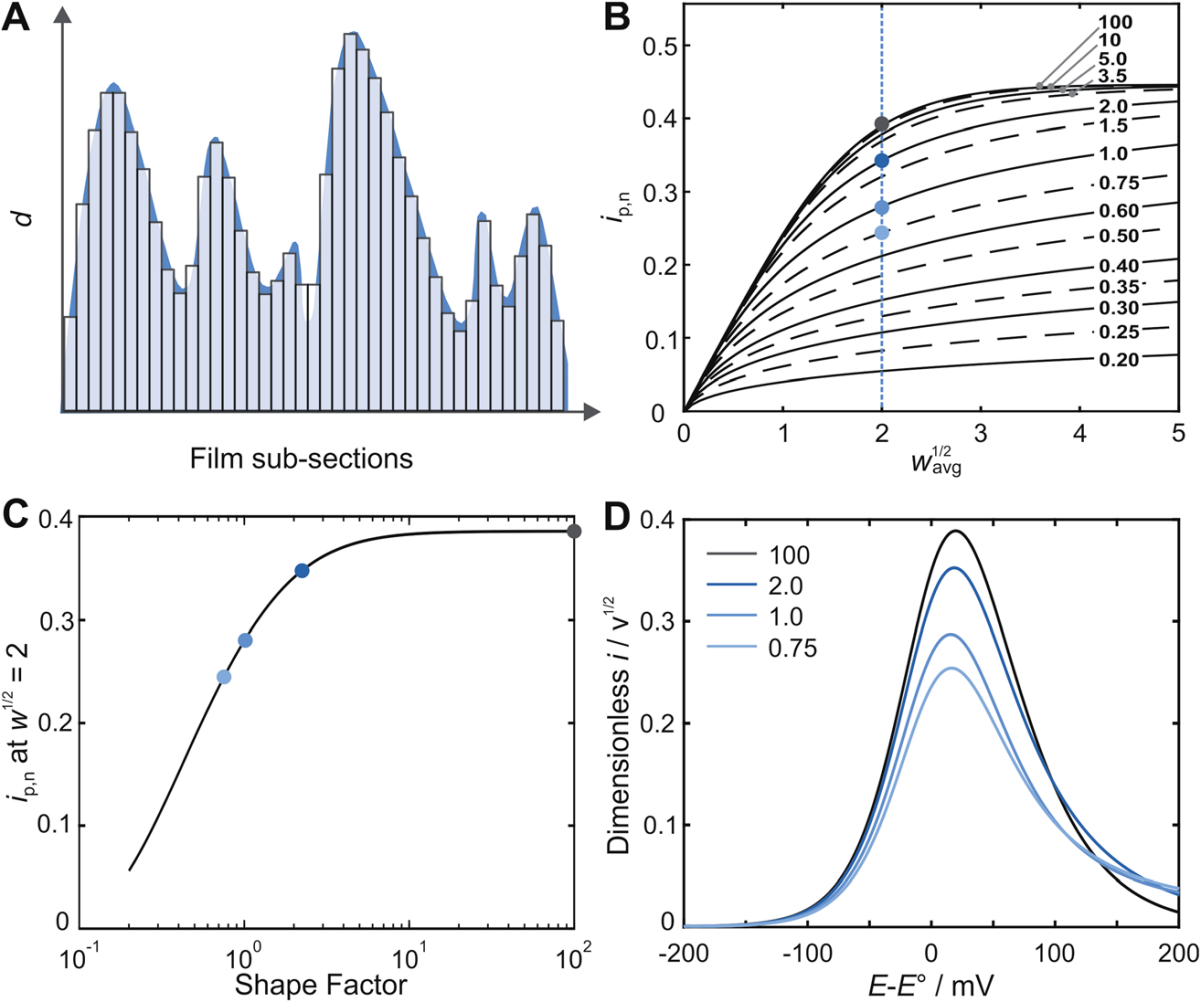
Film thickness distribution determination by the deconstruction method. (A) Schematic illustration showing the “deconstruction” of a rough film (the shape factor is 2.5) into a series of individually independent smooth films. (B) Calculated normalized peak current *vs. w*^1/2^ for a series of shape factor values between 0.20 and 100, showing a systematic deviation from the results for a smooth film (uppermost curve) as the films become increasingly rough (decreasing shape factor values). (C) Normalized peak currents at *w*^1/2^ = 2 (blue dashed line in panel B) plotted *vs.* the Weibull distribution shape factor. This correlation enables for straightforward determination of the thickness distribution from the current response. (D) Predicted LSVs calculated from FEM for a series of shape factors (100, 2, 1, and 0.75) at *w*_avg_^1/2^ = 2. The blue dots in (B) and (C) correspond to the normalized peak current from the LSVs shown in (D).

A straightforward approach for the prediction of the peak current for a rough film is to “deconstruct” it into a series of independent and perfectly smooth film sub-sections (Fig. 2A).

The underlying assumption of planar (one-dimensional) electron transfer within each sub-section allows for the use of an algebraic equation for the peak current for a smooth film (eqn (S3)†) for each of the sections, and then for the total current to be determined by taking the average. The theoretical electrochemical response of a perfectly smooth film in an LSV experiment was previously solved analytically using the Laplace Transform technique, resulting in integral equations for the theoretical LSV current–potential curves.^22^ This includes an algebraic expression for the peak current *vs. w*^1/2^ (eqn (S2)†), with a stated accuracy of 0.5% when compared to the results obtained from the evaluation of the integral equations. An algebraic equation with tighter agreement was needed for this work. Therefore, an updated algebraic equation for peak current was obtained (see details in ESI Section S2†).

### The finite element method for the time and space dependent concentration profiles

The finite element method^24^ (FEM), was used by means of the Matlab® Differential Equations Toolbox to account for edge effects and non-planar diffusion. In contrast to the deconstruction method, in which the film is represented as a series of independent sections, the finite element method treats the entire film as one complete piece. In this approach, the time and space-dependent concentration profiles within the film are determined by solution of a partial differential eqn (3), where the value for *w*^1/2^ from the problem for smooth films is replaced by *w*_avg_^1/2^ according to eqn (4), because the characteristic film thickness (*d*) is now the average film thickness (*d*_avg_):3
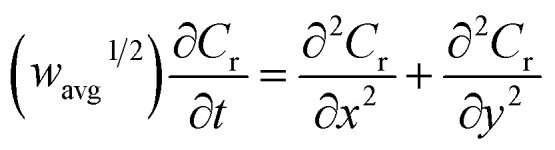

4
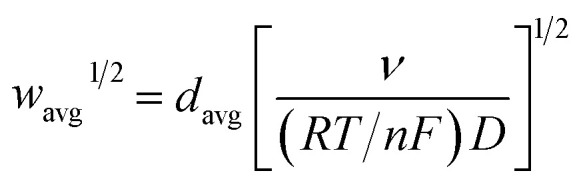

5
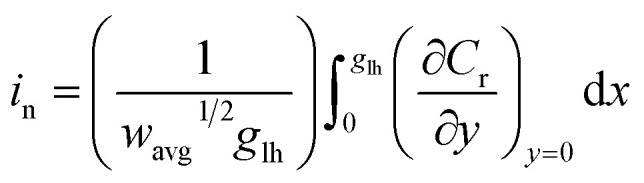



For each time point, the current was calculated by evaluating the concentration gradient at the electrode surface (*C*_r_ is the concentration of the reduced form of the redox species within the film) and integrating the result according to eqn (5), where (*g*_lh_) is the geometric length to height ratio of the film. The complete dimensionless formulation of the problem, as well as additional details regarding the finite element method implementation are included in ESI Sections S3 and S4.†

## Results and discussion

### Normalized peak current plots for films of increasing inhomogeneity in their thickness

Prediction of the peak current for a given experimental time scale was carried out with the deconstruction method (Fig. 2A), using 50 000 film subsections, for a series of shape factors in the range of 0.2–100. The resulting *i*_p,n_*vs. w*_avg_^1/2^ plot (Fig. 2B) displays a similar trend for all shape factors. At low *w*_avg_^1/2^ values, *i*_p,n_ is proportional to *w*_avg_^1/2^. This linear region corresponds to the time scales for which the electrons can reach all boundaries of the film (*δ* ≫ *d*, *δ*_3_ in Fig. 1). The extent of the linear region depends on the shape factor, but within the linear region, the peak currents at high *w*_avg_^1/2^ values are identical for all shape factor values. At high *w*_avg_^1/2^ values, *i*_p,n_ is independent of *w*_avg_^1/2^ and also identical for all shape factor values. This plateau region corresponds to the time scale for which the electrons do not reach any of the boundary (*δ* ≪ *d*, *δ*_1_ in Fig. 1). The transition between the linear and plateau regimes holds the information on the relative positions of the boundaries (*δ* ≈ *d*, *δ*_2_ in Fig. 1), *i.e.* the film thickness distribution. The *i*_p,n_ values deviate from the linear region sooner and reach the plateau later as the shape factor decreases because the first boundaries are reached sooner and the last boundary is reached later as the film inhomogeneity increases. This trend is clearly visible for films with shape factors from 100 to 3.5 in Fig. 2B. The *i*_p,n_ values also eventually reach the plateau for all other shape factors at high *w*_avg_^1/2^ values.

The substantial changes observed in the transition region, where the diffusion layer passes through the roughness features of the film, allow for the use of the *i*_p,n_*vs. w*_avg_^1/2^ plot for deducing the film thickness distribution based on experimental data. The thickness distribution can then be obtained from the shape factor of the theoretical *i*_p,n_*vs. w*_avg_^1/2^ plot matching the experimental *i*_p,n_*vs. w*_avg_^1/2^ plot. In order to make the shape factor determination more accurate, a correlation was constructed, using the dimensionless peak current value at a reference line at *w*_avg_^1/2^ = 2 (Fig. 2C). In addition, FEM was used to calculate the entire LSVs for visualization of the effect of the shape factor (Fig. 2D). Besides the peak current that decreases as the shape factor decreases, the shape of the entire LSV also changes when varying the shape factor when all other parameters are held constant.

### Quantitative evaluation of hemispherical diffusion contributions

**Fig. 3:**
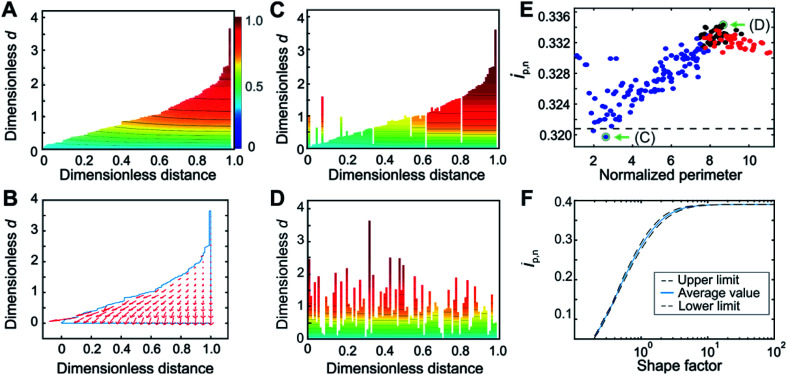
Film thickness distribution determination by the Finite Element Method. (A) Evidence for the presence of hemispherical diffusion from the curvature in the concentration profile and (B) from the concentration gradient flow profile for a film with sub-thicknesses arranged in an ascending order. (C) Concentration profile for the arrangements that minimize and (D) maximize the contribution from hemispherical diffusion. (E) Normalized peak currents for various arrangements of 100 film sub-sections shuffled randomly, starting from either the most ordered arrangement (blue points), the most disordered arrangement (red points), or from the initial random arrangement (black points), in order to cover the entire range of possible degrees of disorder. The perimeter for each arrangement is normalized with respect to the minimum possible perimeter. The deconstruction result is shown as a dashed black line. (F) Correlation between the normalized peak currents and the Weibull distribution shape factor obtained from FEM in which deconstruction was used as an internal standard. Arrangements corresponding to the minimum and maximum of the plot in (E), shown in (C) and (D) respectively, and highlighted with green arrows in (E), were used for the construction of the upper and lower limits of the correlation shown in (F) as black dashed lines; the solid blue line is the average of these two results. An enlarged version of panel (F) is given in Fig. S11.† For panels (A–E), the shape factor was 1.5. Counter-ion transport is assumed to be non-limiting.

Although the calculation procedure afforded by the deconstruction method is especially fast and convenient for obtaining the normalized peak current variation as a function of normalized scan-rate (*i*_p,n_*vs. w*_avg_^1/2^ plot), the accuracy of the resulting film thickness distribution is bound to the assumption of planar electron diffusion through the film. In order to quantitatively account for the contribution of hemispherical electron diffusion, the finite element method (FEM) was used for generating the concentration profiles and gradients for a film thickness distribution with a shape factor of 1.5 (Fig. 3). This shape factor was selected because the effects of hemispherical diffusion were greatest at this value, according to the comparison of deconstruction and FEM results for the entire range of Weibull distribution shape factors (see details in ESI Section S5.2†).

The film sub-sections were firstly arranged in an ascending order to reveal insights into the contour line curvature. For the case of planar electron transfer, the contour lines of the concentration profile would be entirely flat throughout the film. Instead, the concentration profile for the ordered arrangement (Fig. 3A) revealed curvature, which can be attributed to contributions from hemispherical diffusion. This was confirmed by the non-planar direction of the electron flow depicted in the flow profile (Fig. 3B). Moreover, comparison of the concentration profile from an arrangement having a strong planar diffusional character (Fig. 3C) with the one from a highly disordered arrangement having strong hemispherical character (Fig. 3D) showed substantial differences in their curvatures. This qualitatively demonstrates that film sub-section arrangements have an impact on the resulting electron-transfer within the film. This implies that the various possible arrangements of the film sub-sections for a single thickness distribution affect the resulting normalized peak currents from which the shape factor is extracted.

For a quantitative evaluation of the effect of film sub-section arrangement on the resulting hemispherical diffusion, a set of 100 sub-sections for a film with a shape factor of 1.5 was generated and shuffled randomly between two extreme configurations containing minimum and maximum disorder (Fig. 3E). The objective was to identify the minimum and maximum *i*_p,n_ values obtained for a given shape factor as a function of the arrangement and, by extension, the minimum and maximum thickness distribution values related to these *i*_p,n_ values. The normalized peak currents were calculated and plotted *versus* the normalized total perimeter, which was used as a general measure of film sub-section disorder. The minimum *i*_p,n_ value (0.320) was obtained from a cluster of values with a perimeter ratio of 2.6, while the maximum *i*_p,n_ (0.334) was derived from a cluster of values with a perimeter ratio of 8.7. The minimum value, being closest to the deconstruction result (black dashed line in Fig. 3E), represents the film configuration that corresponds to a condition of mostly planar diffusion, and the maximum value represents the film configuration that corresponds to a maximum contribution from hemispherical diffusion.

Through these calculations, the impact of hemispherical diffusion on *i*_p,n_ was quantified, allowing for the calculation of a correlation between *i*_p,n_ and shape factor based on FEM results that includes lower and upper confidence limits. For the calculation of these limits, one representative configuration from the minimum and maximum value clusters was identified (Fig. 3C and D). Then, for each shape factor, the sampled sub-sections were rearranged according to these limiting configurations before calculation of the *i*_p,n_ values by FEM, and the difference between this result and the deconstruction result (which was used as an internal standard that depends on planar diffusion only), was used to add lower and upper limits to the correlation (Fig. 3F). The average of the two results is reported as the center line. Probability distribution functions (PDFs) at the lower and upper limits at three points in the correlation (nominal shape factor = 0.5, 1.5, and 7.0) were compared and did not look substantially different (see ESI Section S5.3†). This means that although the film sub-section arrangement impacts the peak current and thus the resulting film thickness distribution value, it remains relatively minor.

While it is possible to determine the shape factor (and therefore the distribution) using only the value of *i*_p,n_ at *w*_avg_^1/2^ = 2 (Fig. 3F), a peak current overlay plot similar to that of Fig. 2B, but which uses FEM and deconstruction was calculated (Fig. S9†) to enable extraction of the shape factor using *i*_p,n_ values at any *w*_avg_^1/2^ values in the transition region. This is useful when the exact value of *i*_p,n_ at *w*_avg_^1/2^ = 2 is not available experimentally (the exact value of the scan rate corresponding to *w*_avg_^1/2^ = 2 cannot be predicted beforehand since the value of *d*_avg_ is typically unknown, see eqn (4). In Fig. S9,† deconstruction was used for shape factors below 0.75 and FEM was used for shape factors 0.75 and greater. This was possible because the average FEM values (when using deconstruction as an internal standard) were the same as the deconstruction values for shape factors below 0.75 (see ESI Fig. S8 and ESI Section S5.4†). Physically, this means that the effects of hemispherical diffusion are negligible for shape factors below 0.75.

### Experimental example and comparison with AFM

**Fig. 4:**
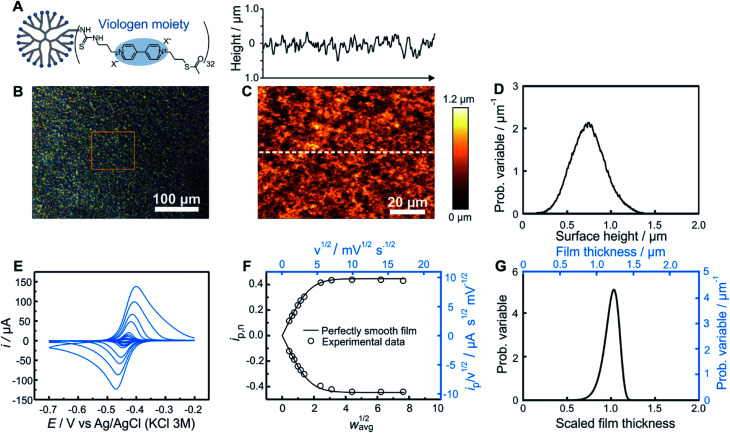
Comparison of electrochemistry and AFM results for a smooth dendrimer film. (A) Structure of the viologen modified dendrimer used for film assembly by means of drop-casting. (B) Optical microscope image with the approximate area imaged by AFM framed in orange, (C) AFM image (bottom panel) with line scan (upper panel), and (D) resulting surface height distribution based on AFM (see ESI Section S1.1† for definition of the probability variable). (E) Linear sweep voltammograms at increasing scan rates (from 1 to 300 mV s^–1^). (F) The corresponding normalized peak current plot generated using the peak currents from the LSVs in (D) (open dots) and theoretical *i*_p,n_*vs. w*_avg_^1/2^ curve for a perfectly smooth film (solid line). The plot is depicted with both dimensionless (bottom *x* axis and left *y* axis, black) and dimensional (top *x* axis and right *y* axis, blue) axes. (G) Normalized film thickness distribution resulting from the LSV measurements (bottom *x* axis and left *y* axis, black) and the corresponding dimensional film thickness distribution obtained by multiplying with the film thickness (top *x* axis and right *y* axis, blue). The experimental data are from ref. 20.

**Fig. 5:**
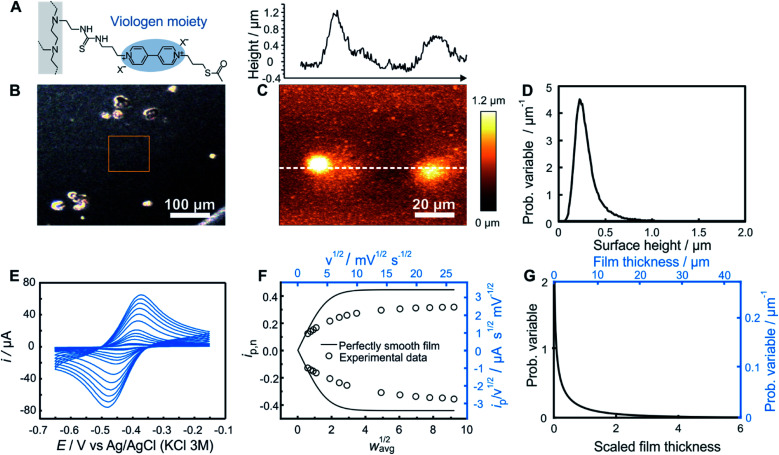
Comparison of electrochemistry and AFM results for a rough polymer film. (A) Structure of the viologen modified polymer used for film assembly by means of drop-casting. (B) Optical microscope image with the approximate area imaged by AFM framed in orange, (C) AFM image (bottom panel) with line scan (upper panel), and (D) resulting surface height distribution based on AFM (see ESI Section S1.1† for definition of the probability variable). (E) Linear sweep voltammograms at increasing scan rates (from 2 to 700 mV s^–1^). (F) The corresponding normalized peak current plot generated using the peak currents from the LSVs in (D) (open dots) and theoretical *i*_p,n_*vs. w*_avg_^1/2^ curve for a perfectly smooth film (solid line). The plot is depicted with both dimensionless (bottom *x* axis and left *y* axis, black) and dimensional (top *x* axis and right *y* axis, blue) axes. (G) Normalized film thickness distribution resulting from the LSV measurements (bottom *x* axis and left *y* axis, black) and the corresponding dimensional film thickness distribution obtained by multiplying with the film thickness (top *x* axis and right *y* axis, blue). The experimental data are from ref. 20.

As an experimental example showing the usefulness of the electroanalytical approach, as well as its complementarity with AFM, both methods were applied for the characterization of redox-active films assembled from viologen modified macromolecules. We compare previously reported data^20^ for smooth films (Fig. 4) assembled from the drop-casting of viologen-modified dendrimers (Fig. 4A) as well as for rough films (Fig. 5) made from the drop-casting of viologen-modified polymers (Fig. 5A).

Optical microscopy of the smooth film revealed surface homogeneity over a large area (Fig. 4B), which was confirmed by AFM on a smaller sampled area (Fig. 4C). The probability distribution function of the surface height (Fig. 4D) and roughness parameters were calculated using Gwyddion software.^25^ The average value of the surface height is 0.75 μm and the root mean square roughness is 0.20 μm. For electrochemical determination of the thickness distribution, the peak currents were extracted from both the anodic and cathodic scans of the cyclic voltammograms obtained for the same redox film at various scan rates (Fig. 4E). The normalized dimensionless peak values obtained according to eqn (2), are plotted against the normalized scan-rates obtained according to eqn (4) (Fig. 4F). The experimental data are in close agreement with the theoretical *i*_p,n_*vs. w*_avg_^1/2^ plot (black solid trace) expected for a perfectly homogenous film. Using the *i*_p,n_ value at *w*_avg_^1/2^ = 2, a Weibull distribution shape factor value of 14 was directly determined based on the correlation given in Fig. 3F and was used to generate the probability distribution function (see ESI Section S1.1†) shown in Fig. 4G. The relative standard deviation for the thickness distribution of the electroactive fraction of the film obtained directly from the shape factor (Fig. S1†) was 9%.

In the case of this smooth film, both of the limiting regions (the linear dependence at low scan rates and the plateau at high scan rates) are experimentally accessible (Fig. 4F). Information from the two limiting regions (the slope at low scan rates and the plateau value at high scan rates) were used for the determination of two combinations of variables (*d*_avg_/*D*^1/2^ and *CD*^1/2^) which can be used to convert the *i*_p_/*ν*^1/2^*vs. ν*^1/2^ plot to the normalized plot of *i*_p,n_*vs. w*_avg_^1/2^ (see ESI Section S6.1†). This means that the peak currents from the voltammetric measurements alone directly enable the determination of the film thickness distribution without the need to determine any of the individual parameters used for normalization of the peak current and of the scan rate. An additional benefit of the independence of the film thickness distribution from film parameters such as *D*, *d*_avg_, and *C* is that the method is independent of the electrolyte composition which can impact the values of those same parameters.

Knowledge of *D*, however, is useful for extracting the absolute film thickness *d*_avg_ from the electrochemical data according to a previously reported method^14^ or more conveniently from the intersection of the extrapolated plateau and linear regions in Fig. 4F (see ESI Section S6.2†). In the case of this particular film based on redox-active dendrimers, the value of *D* was determined previously.^20^ The resulting *d*_avg_ value extracted from the electrochemical data is 1.2 μm, and was used to dimensionalize the probability distribution function of the film thickness (Fig. 4G). To allow for a direct comparison with AFM surface measurements, the film thickness distribution can be further converted to a dimensional surface distribution (see ESI Section S1.3†). The resulting average surface height value (0.88 μm) and the root mean square roughness (0.10 μm) are in good agreement with the corresponding values obtained from AFM considering the different characterization conditions (dry *vs.* solvated state).

One additional parameter of interest is the resolution of the electrochemical method. This was determined by analyzing the variations of the electrochemistry derived film thickness distributions (relative standard deviations). After preparation of a single smooth film, a series of nine successive CVs (Fig. S10†) were taken at a scan rate corresponding to *w*_avg_^1/2^ = 2 for this film preparation (*ν* = 10 mV s^–1^). Data treatment for each CV was then performed separately, resulting in a series of nine individual shape factor determinations with their corresponding normalized RSD values. The standard deviation of these nine RSD values was found to be 1% (Table S1†).

In the case of rough films (Fig. 5) with very low shape factors, the two limiting regimes for the experimental *i*_p,n_*vs. w*_avg_^1/2^ plot may not be accessible. This is illustrated with the example based on a film fabricated through drop-casting of a viologen modified polymer. Both the optical (Fig. 5B) and AFM (Fig. 5C) images revealed the presence of large polymer aggregates. The probability distribution function of the height features on the surface was subsequently extracted using Gwyddion software^25^ (Fig. 5D). The average value of the surface height is 0.31 μm and the root mean square roughness is 0.17 μm.

The cyclic voltammetry measurements of this same film (Fig. 5E) were used for extraction of the peak currents and for construction of the experimental *i*_p,n_*vs. w*_avg_^1/2^ plot (Fig. 5F). Both the linear region and the plateau were not accessible experimentally *via* the scan rate. Nevertheless, the transition region was sufficient for the determination of the film thickness distribution because the normalization of *i*_p,n_ and *w*_avg_^1/2^ can be performed independently of these regions. In such a case, the values of *D*, *d*_avg_, and *C* which are needed for normalization must be determined individually from separate experiments. The value of *D* for films made of the same redox polymer was determined previously^14^ and was used to determine *C*. The value of *d*_avg_ (7.32 μm) was obtained from *C* and the surface concentration in viologen according to a previously reported procedure.^14^,^20^

The dimensionless normalized *i*_p,n_*vs. w*_avg_^1/2^ plot (Fig. 5F) constructed based on *D*, *d*_avg_, and *C* values was used for extraction of the value of *i*_p,n_ at *w*_avg_^1/2^ = 2, from which a Weibull distribution shape factor of 0.60 was directly determined, using the correlation given in Fig. 3F. The probability distribution function corresponding to the determined shape factor was then generated (Fig. 5G) as described in ESI Section S1.1.† The relative standard deviation for the film thickness distribution obtained from the shape factor using the correlation in Fig. S1† was 176%. To allow for a direct comparison with AFM results, as in the case of the dendrimer, the dimensional film thickness distribution was generated (Fig. 5G) and then converted into a surface distribution (see ESI Section S1.3†). The average surface height value (7.32 μm) and the root mean square roughness (12.87 μm) are substantially different from the corresponding values obtained from AFM. This discrepancy is attributed to the selection of a relatively smooth region for AFM imaging (orange frame in Fig. 5B) in comparison to the large aggregates in other regions of the same sample as observed in the optical image (Fig. 5B).

This result highlights an important advantage of the electrochemical method in that it is naturally an ensemble method, whereby the entire surface is sampled as opposed to a small subsection. Although direct observation of the surface by AFM or optical methods is highly desirable, in particular, for its ability to show the sizes and spatial locations of aggregates, the results are highly dependent on the selection of the sampling area (typically only up to 100 μm × 100 μm). Conversely, although the electrochemistry measurements are representative of the entire surface, the exact locations and geometries of the aggregates are not available. This emphasizes the complementary nature of the two methods and highlights the unique information derived from electrochemistry.

Moreover, the electrochemical method measures from the bottom of the film at the electrode surface upwards through the film. Therefore, it naturally probes the film thickness distribution, in contrast to AFM which directly gives surface roughness information but requires scratching of the sample to access thickness information. Furthermore, while AFM can be performed on solvated samples or in the presence of electrolytes, soft samples such as the hydrogel films used in the present study can be particularly challenging to image.^26^ The electrochemical method, in contrast, is intrinsically performed in the presence of the electrolyte, which correspond to the operational conditions for the applications of the film. Therefore, optimization of the solvent composition and the evaluation of solvent effects is convenient when using the electrochemical method.

Although the method presented in this work is broadly applicable to redox-active films, the limitations of the scope of this model should be noted. Firstly, the time scale of the electron transfer within the film needs to be sufficiently slow so that the diffusional regime is accessible within the time scale of the linear sweep voltammetry experiment. In other words, diffusion layer thicknesses with dimensions in the range of the thicknesses of all film sub-sections must be accessible *via* the scan rate (see eqn (1)). Generally, films in which electrons are transported by diffusional or “electron-hopping” mechanisms are covered by this model. Secondly, the accuracy of the peak current analysis depends on the ease of subtraction of the baseline related to the capacitive current. In particular, possible interferences may arise from non-uniform capacitance (*i.e.* potential dependent capacitance at the underlying electrode) that would distort the cyclic voltammograms and therefore adversely affect the peak current analysis. Additionally, since the model assumes electrochemical reversibility for the electron transfer at the electrode/film interface, peak currents from films that have slow heterogeneous electron transfer rates cannot be analyzed with the current version of the model.

In general, the design requirements of redox active films for electrocatalytic applications include high loading of the redox moiety (catalyst and/or electron relays) and fast charge transfer at the electrode interface, implying that the peak to baseline ratio is significant and that the reversibility condition is usually fulfilled, which makes this method useful for the characterization of a broad range of modified electrodes. Moreover, the analysis of films that display significant and non-uniform capacitive current and/or slow heterogeneous charge transfer can in principle also become accessible by adapting the model to the analysis of the current response obtained by potential step voltammetry. This method would in principle also enable characterization of very thick films that are not conveniently accessible in the time scale of linear sweep voltammetry.

Future development of the method may also take advantage of the counter ion transport associated with the electron transport, purposely making the former limiting to deliver the arrangement of the surface features of the films, which is currently not accessible *via* the electrochemical method. As a final note, pin-holes which do not enable any electron transport are not included in the scope of this work. In this case, methods are available which make use of rotating disk electrodes with variable rotation rates^27^ or scanning electrochemical cell microscopy.^28^

## Conclusions

In this work, a novel electrochemical method based on linear sweep voltammetry was developed as a highly complementary method to AFM for the characterization of electroactive films. By measuring the peak currents in a series of linear sweep voltammograms, going from low to high scan rate, the underlying film thickness distribution in terms of the Weibull distribution shape factor can be determined from a plot of the normalized peak current *vs.* the experimental time scale. From the shape factor, the probability distribution function can be constructed as a quantitative description of the film thickness distribution. As an “ensemble” method, it samples the complete surface and is therefore not sensitive to the location of the sampling area. Furthermore, as an electrochemical method, it focuses on the electroactive portion of the film on the electrode, which is of particular relevance to films which are actively participating in redox or electro-catalytic processes. Finally, this information is provided in the solvated state which is relevant for the final working conditions of the modified electrode. This is ultimately useful for the optimization of the performance of these films, in which the film thickness is a critical parameter, defining the catalytic or light-induced current output, and the fraction of catalyst effectively contributing to the reaction.

## Experimental section

### Materials and methods

Unless stated otherwise, all reagents used in experiment were purchased from Sigma-Aldrich. All the materials were directly used as received without further purification.

### Film preparation

The preparation and characterization procedure for the redox-active films used to illustrate the application of the present method for determination of the thickness distribution was previously reported in detail.^20^ We recall here the film preparation corresponding to the specific data used in the present study: viologen modified dendrimer^18^ (200 μg cm^–2^) was cast from a 2 μL droplet onto a gold electrode (2 mm diameter) with 0.5 μL Tris-buffer (0.1 M, pH 9.0), and allowed to dry in a closed container at room temperature under a water saturated atmosphere for 24 h. The electrode was then dried in the air for another hour. Viologen modified polymer^10^ (200 μg cm^–2^) was cast from a 2.5 μL droplet onto a glassy carbon electrode (3 mm diameter), and dried using the same conditions as that of the dendrimer.

### Electrochemical characterization

Cyclic voltammograms were performed in phosphate buffer (0.1 M, pH 7.2) under anaerobic conditions at room temperature using Gamry Potentiostats and an Autolab PGSTAT12 Bipotentiostat. A platinum wire and Ag/AgCl/3 M KCl were used as the counter and reference electrodes, respectively. Before preparation of the characteristic normalized peak current plot, interactions were accounted for using slow scan-rate LSVs, in which the experimentally determined peak currents were converted to their Langmuir equivalents (see details in ESI Section S7†).

### AFM characterization

The AFM measurements were conducted in the AC mode by NanoWizard 3 (JPK) with cantilever of the type NSC15 (MikroMasch).

## Conflicts of interest

There are no conflicts to declare.

## Supplementary Material

Supplementary information
