# Supplementary material for: The electron as a probe to measure the thickness distributions of electroactive films
Source: Chem Sci. 2019 Nov 18;11(4):937–46. doi: 10.1039/c9sc03653a (PMC7583368; doi:10.1039/c9sc03653a)
Supplement: Supplementary file 1 [file SC-011-C9SC03653A-s001.pdf]

Electronic Supplementary Material (ESI) for Chemical Science.
This journal is © The Royal Society of Chemistry 2019

Supporting Information

The Electron as a Probe to Measure the Thickness Distributions of Electroactive Films

Darren Buesen, Huaiguang Li, and Nicolas Plumeré*

Center for Electrochemical Sciences (CES), Faculty of Chemistry and Biochemistry, Ruhr University Bochum, Universitätsstr. 150, D-44780 Bochum, Germany

E-mail: nicolas.plumere@rub.de

Table of Contents

S1. Film thickness distribution and surface roughness calculations 2
S1.1. Probability distribution function 2
S1.2. Relative standard deviation 3
S1.3. Root mean square roughness 3
S2. Development of the algebraic equation used in deconstruction 4
S2.1. Calculation of LSVs from an integral equation 4
S2.2. Curve fit comparison 5
S2.3. Fitting equation for use in deconstruction 6
S3. Boundary value problem scaling and conditions 7
S3.1. Variable scaling and ranges 7
S3.2. Boundary and Initial conditions 7
S4. Finite element mesh optimization and verification 8
S5. Shape factor correlation upper and lower limits from limiting configurations 10
S5.1. Perimeter ratio as a quantitative measure of film disorder 10

S5.2. Focus on SF=1.5 based on screening with the ascending ordered configuration......................11
S5.3. Evaluation of arrangement on the resulting film thickness distributions.................................13
S5.4. Average FEM and deconstruction shape factor correlation curve comparison 14
S5.5. Method resolution...16
S6. Simplified procedure when slope and plateau are experimentally accessible..............................17
S6.1. Simplified non-dimensionalization 17
S6.2. Average thickness from transition scan rate 18
S7. Accounting for interactions 19
S8. Enlarged shape factor correlation plot with upper and lower limits..20
S9. Table of symbols..21
S10. References 22

S1. Film thickness distribution and surface roughness calculations

For calculations based on AFM images, Gwyddion[1] software was used. For calculations based on the electrochemical method, the one-parameter Weibull distribution[2] was used (scale factor = 1, shape factor = variable). Matlab was used for statistical sampling.

S1.1. Probability distribution function

The dimensional probability distribution function (PDF) based on an AFM image is reported in the "calculate 1D statistical functions" section of Gwyddion[1] after importing the image and setting the minimum value to zero.

The dimensionless probability distribution function (PDF) is obtained by use of the Matlab built-in function "wblpdf". The mean value of the film thickness (d_{avg}) is then used to convert it to the corresponding dimensional PDF (multiplying the x axis by d_{avg} and dividing the y axis by d_{avg}).

S1.2. Relative standard deviation

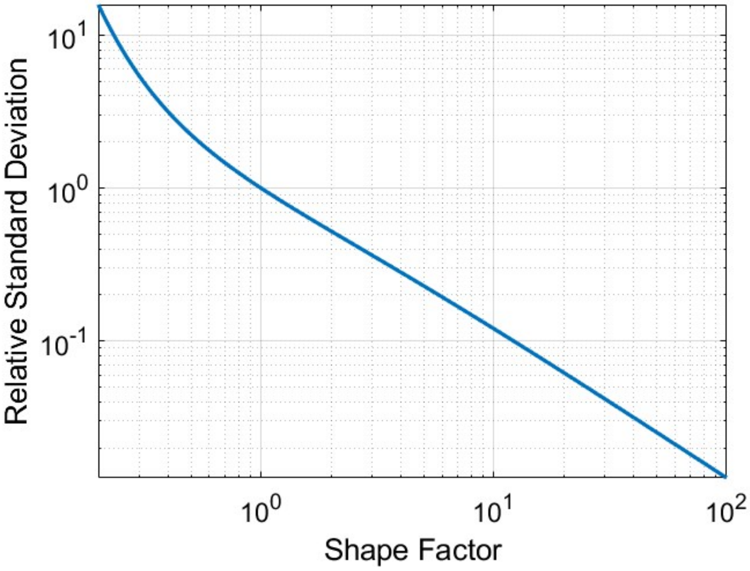

Figure S1. Correlation of the relative standard deviation (RSD) for shape factors in the range of 0.2 to 100. The correlation was calculated by making use of previously reported analytical expressions[2].

S1.3. Root mean square roughness

The root mean square (RMS) roughness based on an AFM image is reported in the "statistical quantities" section of Gwyddion[1] after importing the image and setting the minimum value to zero.

For calculation of the RMS roughness from the electrochemical method, a set of film thicknesses is firstly generated by using the Matlab built-in function "wblpdf" and then scaled so that the average thickness of the distribution is equal to the experimentally determined value. Then, the film thickness distribution is converted to a surface distribution by subtracting all film sub-thicknesses by the minimum value of the film thickness distribution. Lastly, the average surface height (mean line) is calculated, and then used for calculation of the root mean square roughness by comparison of the deviations (y_i values) of the surface distribution from the mean line[3].

S2. Development of the algebraic equation used in deconstruction

S2.1. Calculation of LSVs from an integral equation

In the case of perfectly smooth redox-active films, a previous publication[4] reported on the calculation of the linear sweep voltammograms (LSVs) from an integral equation, and the extraction of the peak currents to formulate an algebraic expression for the normalized peak current as a function of $w^{1/2}$. Replication of the solution of the integral equation is the first step required for the development of an analytical expression for peak current with higher accuracy. The obtained result, shown in Figure S2, is in agreement with Figure 1 in reference [4].

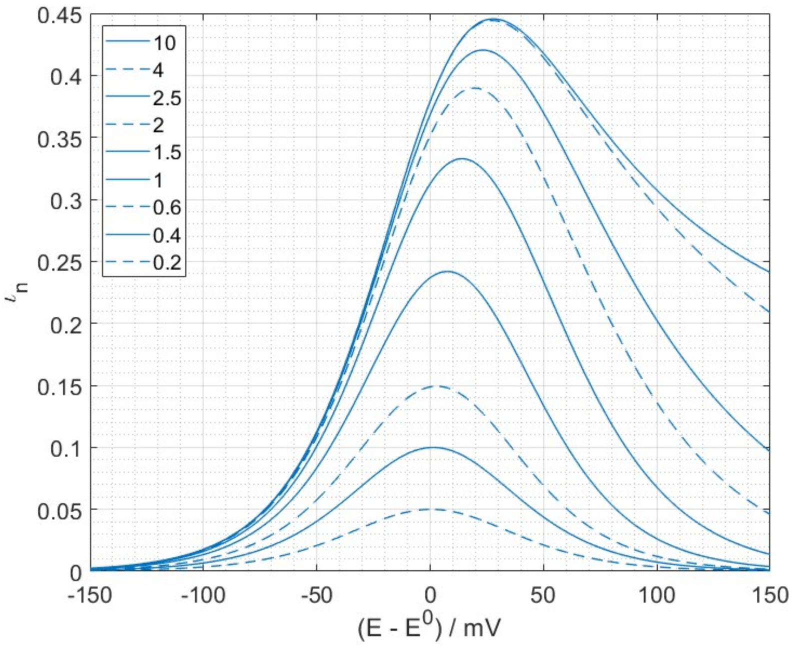

Figure S2. LSV overlay plot of normalized current i_n obtained by the solution of an integral equation[4] for increasing $w^{1/2}$ values (0.2, 0.4, 0.6, 1, 1.5, 2, 2.5, 4, and 10). The normalized current (i_n) is plotted *versus* the applied electrode potential referenced to the standard redox potential E^0 of the redox active couple in the film.

S2.2. Curve fit comparison

After calculation of the LSVs by use of the integral equation, the normalized peak current values ($i_{p,n}$) for $w^{1/2}$ values were then fitted according to a rational polynomial using the Matlab curve fit tool, resulting in Equation S1. The closeness of the results with respect to those predicted according to the original curve fit function (Equation S2) are shown in Figure S3. This form of the fitting equation is different from Equation 18 shown in the reference [4], because it is for the calculation of dimensional current densities. However, normalization of the current, substitution of $w^{1/2}$, and taking a basis surface area of d^2, results in Equation S2 for the normalized peak current as a function of $w^{1/2}$.

Unlike the original curve fit equation, which reaches a natural limit as $w^{1/2}$ increases to values greater than 10, the same is not true for the new curve fit. Therefore, the new curve fit equation is defined as a piecewise function, in which the calculated $i_{p,n}$ value based on Equation S1 holds for $0 \leq w^{1/2} \leq 10$, but is set to 0.446 for $w^{1/2} > 10$.

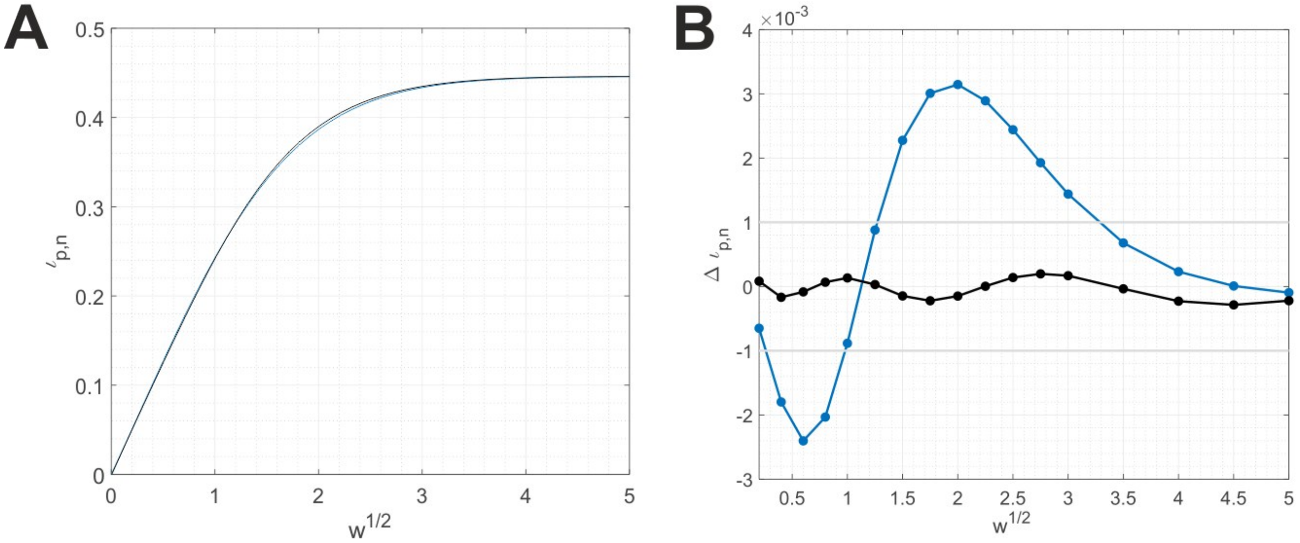

Figure S3. Comparison of algebraic expressions for normalized peak current with results from integral equations for between 0.2 and 5: (A) Overlay plot of the results from equations S1 and S2, and (B) difference plot (the integral equation result minus the respective curve fit result). The expression from reference [3] (blue line in both graphs) is accurate $w^{1/}$ within , whereas the new expression (black line in both graphs) is accurate within .

$$i_{p,n} = \frac{0.4592\,[w^{1/2}]^3 - 0.01716\,[w^{1/2}]^2 + 2.16\,[w^{1/2}] - 0.01071}{[w^{1/2}]^3 + 0.5792\,[w^{1/2}]^2 + 0.8387\,[w^{1/2}] + 8.305} \tag{S1}$$

$$i_{p,n} = 0.446\ tanh\left(0.56\left[w^{1/2}\right] + 0.05\left[w^{1/2}\right]^2\right) \quad \text{(S2)}$$

An overlay plot of the results from the two curve fits is shown in Figure S3. When comparing to the value of the normalized peak at the reference line in the transition region ($w^{1/2} = 2, i_{p,n} = 0.390$), the tolerance of ± 0.001 corresponds to a tolerance of 0.25%. Therefore, use of the algebraic equation developed here allows for a 50% improvement in accuracy when compared to the equation reported in reference [3].

S2.3. Fitting equation for use in deconstruction

In the deconstruction method, an unscaled distribution of film sub-thicknesses is used, unscaled peak currents are calculated for each sub-section, and normalization occurs in the final step in the calculation. Therefore, an algebraic expression for the dimensional peak current is needed. Unscaling of Equation S1 along with the substitution of $w^{1/2}$ results in Equation S3, which was used for the deconstruction calculations.

$$i_p = nFA\left(\frac{C \cdot D}{d}\right)w^{1/2}\left[\frac{0.4592\left[w^{1/2}\right]^3 - 0.01716\left[w^{1/2}\right]^2 + 2.16\left[w^{1/2}\right] - 0.01071}{\left[w^{1/2}\right]^3 + 0.5792\left[w^{1/2}\right]^2 + 0.8387\left[w^{1/2}\right] + 8.305}\right] \quad \text{(S3)}$$

S3. Boundary value problem, scaling and conditions

S3.1. Variable scaling and ranges

The primary variables (concentration, space, scan rate, and time) were scaled according to appropriate reference quantities: $C_r = \bar{C}_r/C$, $g_{lh} = l/d_{avg}$, $x = \bar{x}/(g_{lh}d_{avg})$, $y = \bar{y}/d_{avg}$, $v = \bar{v}/(RT/nF)$, and $t = \bar{t}/t_{ref}$, where $t_{ref} = (RT/nF)/v$.

The ranges of the x, y, and t variables are $(0 \le x \le g_{lh})$, $(0 \le y \le y_{ub})$, and $(0 \le t \le 16)$, respectively. These values are based on a total potential sweep range of $(E^0 - 200\ mV)$ to $(E^0 + 200\ mV)$. The y value at the upper boundary varies according to the film roughness geometry and therefore is denoted as y_{ub}.

S3.2. Boundary and initial conditions

The boundary conditions at the electrode are governed by the Nernst Equation due to the assumption of reversible electron transfer. Combination of the Nernst equation with the linear equation relating potential and time, and an overall material balance on the redox species gives the following boundary condition at the electrode surface as shown in Equation S4.

$$(C_r)_{y=0} = \frac{1}{1 + exp(t - 8)} \quad \text{(S4)}$$

The “no flux” boundary condition $-\nabla C_r = 0$ was applied to the sides and top of the film for the case of two dimensions.

The initial condition is that the film is assumed to be completely in the reduced state at the initial time: $(C_r)_{t=0} = 1$.

S4. Finite element mesh optimization and verification

Just as the algebraic equation used for the calculation of peak currents is critically important of the deconstruction method, and therefore required optimization and

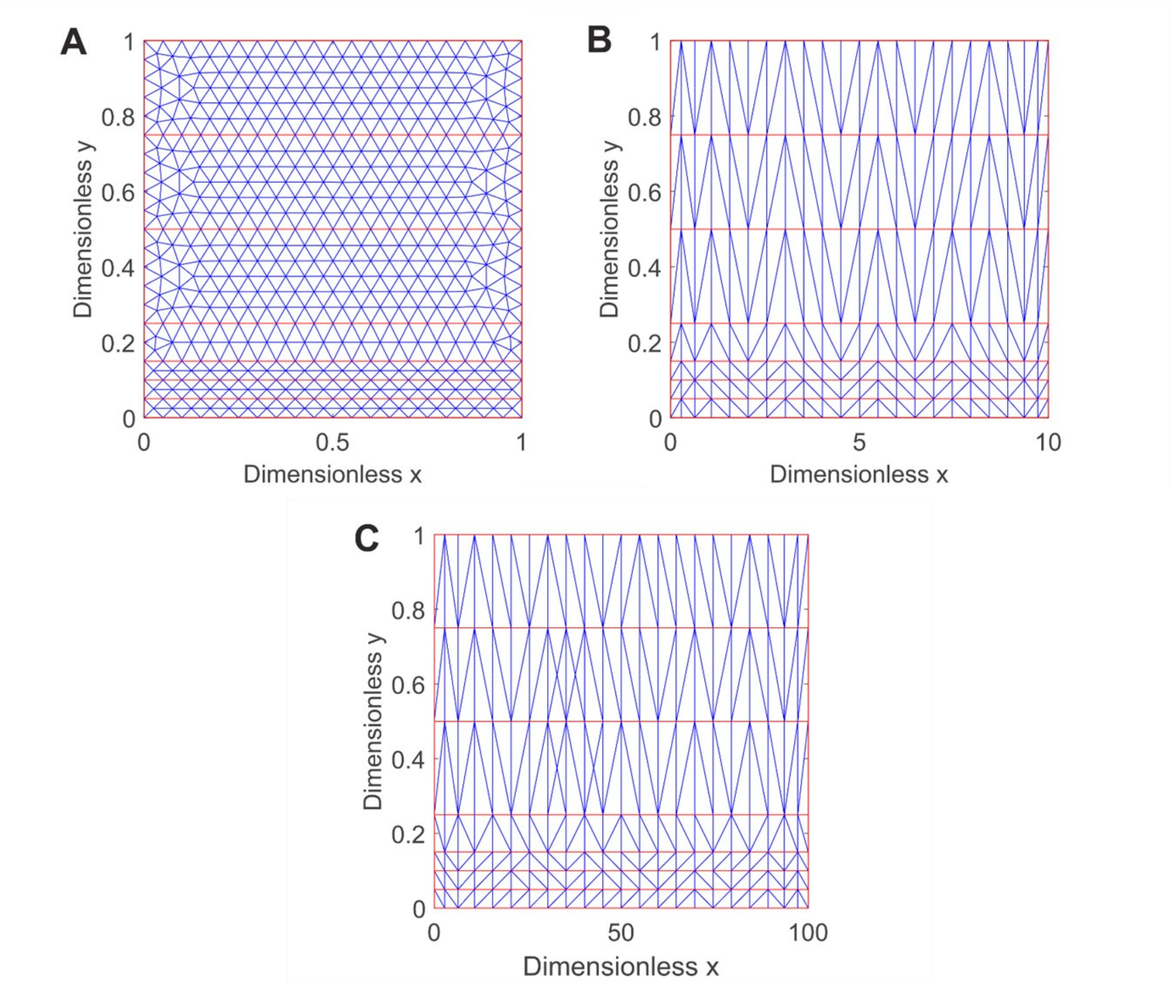

Figure S4. FEM geometry and mesh for a perfectly smooth film with geometric scale factor g_{th} equal to (A) 1, (B) 10, and (C) 100.

verification, the finite element mesh was also optimized and verified.

Since the calculated FEM results are normalized with respect to the length-to-height factor (i.e. g_{lh} appears in the equation for normalized current), the choice of g_{lh} should not change the final result. In order to evaluate the effects of length-to-height ratio for the films shown in Figure S4 with comparable mesh densities, the minimum element length parameter, H_{max}, was scaled according to the value of g_{lh}. Therefore, the corresponding H_{max} values used were 5, 0.5, and 0.05 respectively. For each geometric ratio value, four values for $w^{1/2}$ were chosen which covered the range of interest for $w^{1/2}$ (0.2, 2, 4, and 10), and the $i_{p,n}$ values were compared. The calculated $i_{p,n}$ values for these were all within +/- 0.001 of the corresponding $i_{p,n}$ values calculated from the integral equation for the smooth films problem (0.050, 0.390, 0.445, and 0.446 respectively), verifying the correct implementation of the finite element method for this boundary value problem. Invariability of the results for different g_{lh} factors was also verified. For convenience and consistency, g_{lh} was set to 1 for the subsequent FEM calculations involving rough films.

S5. Shape factor correlation upper and lower limits from limiting configurations

S5.1. Perimeter ratio as a quantitative measure of film disorder

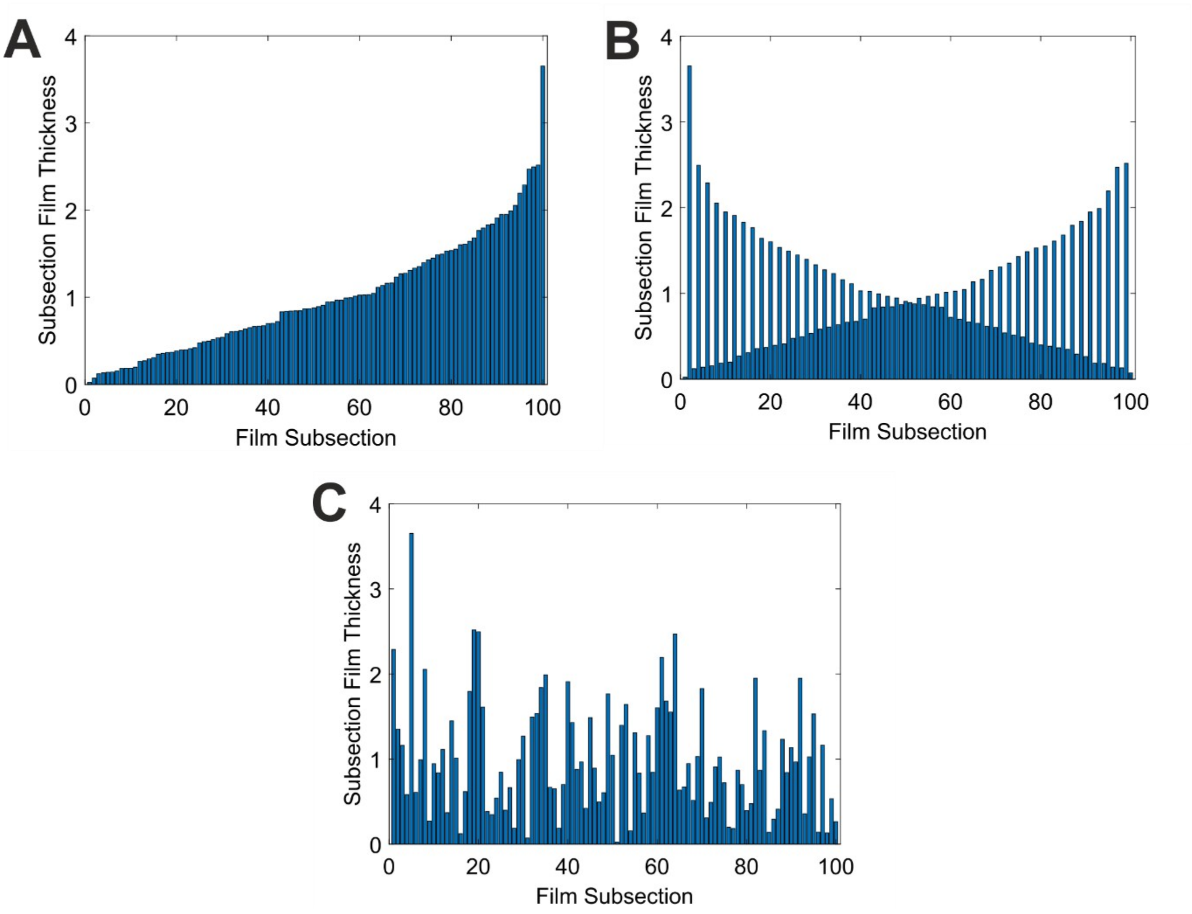

Figure S5. Arrangements (shape factor = 1.5) with variable degrees of disorder, based on total perimeter. The "Ascending Ordered" configuration (A) has the minimum total perimeter, the "Feathered" distribution (B) has the maximum total perimeter, and a typical random arrangement (C) lies within these two extremes. The perimeter ratio of a configuration is defined as its perimeter divided by the perimeter when it is rearranged in the ascended ordered configuration.

S5.2. Focus on SF=1.5 based on screening with the ascending ordered configuration

In order to identify the shape factor at which hemispherical diffusion is greatest, calculations were performed using the ascending ordered configuration. For each shape factor value chosen in the range of 0.2 to 100, a sample size of 100 points was taken, the resulting film sub-sections were arranged in an ascending ordered configuration, FEM and deconstruction calculations were performed in parallel on these identical 100 points, and the difference between FEM and deconstruction results were recorded. For these calculations, the deconstruction method was needed as an internal standard because it gives the normalized peak current for the case of strictly planar diffusion. Therefore, subtracting the FEM and the deconstruction results gives the quantitative amount of hemispherical diffusion for a given shape factor.

Replicates for each shape factor were performed until the standard deviation of the normalized peak current results was within 0.001. Once the hemispherical contribution was calculated at each shape factor, the calculated amount of hemispherical diffusion (obtained from FEM with 100 points sampling and using deconstruction as an internal standard) was then added to the deconstruction result obtained from a higher sampling amount (50,000 points, lower curve in Figure S6) in order to obtain the upper (green) curve in the overlay plot shown in Figure S6.

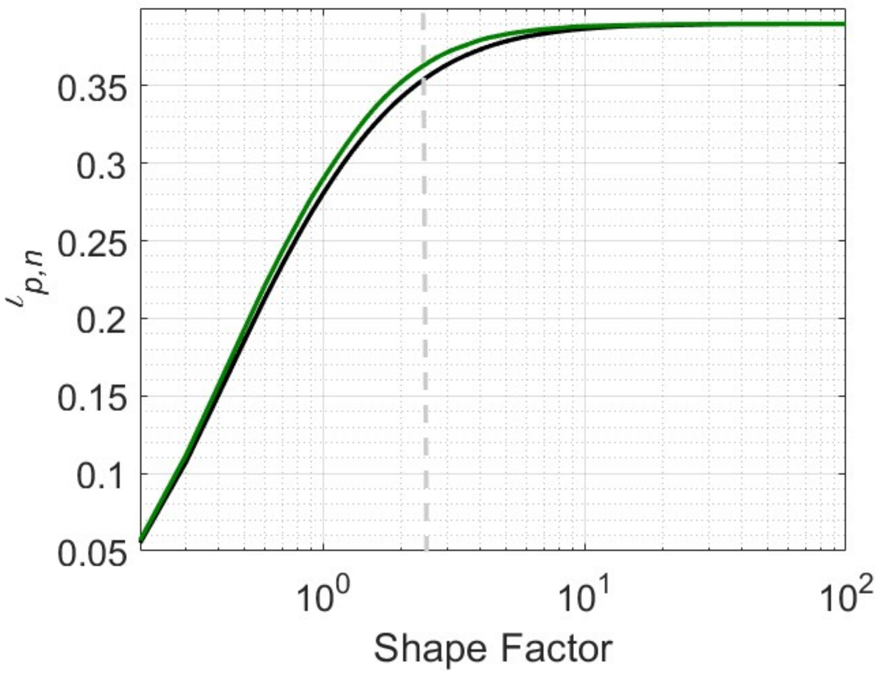

Figure S6. Overlay plot of normalized peak current FEM (green curve) and deconstruction (black curve) results at $w_{avg}^{1/2} = 2$ for identification of 1.5 as the shape factor with maximum hemispherical diffusion (grey dashed line). The ascended ordered distribution was used for the FEM calculations.

The effect of hemispherical diffusion is minimal at the two extremes of film roughness and is maximal at shape factor value of 1.5. Therefore, film sub-thickness distributions with a shape factor of 1.5 were later shuffled for identification of the configurations with the lowest and highest amounts of hemispherical diffusion.

S5.3. Evaluation of arrangement on the resulting film

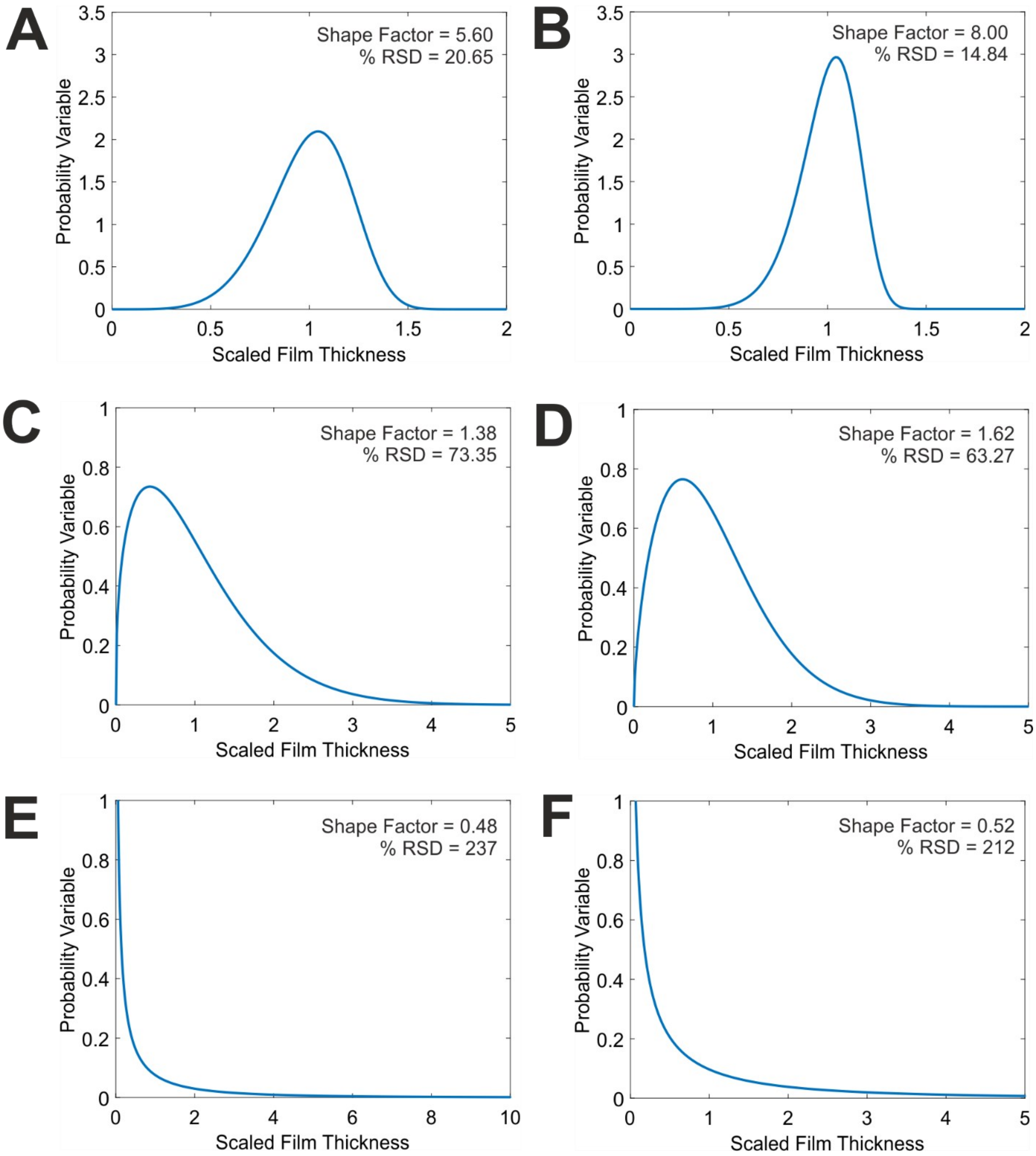

Figure S7. Effect of arrangement on the resulting film thickness distribution functions. Film thickness distributions corresponding to the upper and lower limits of FEM shape factor correlation results based on nominal shape factor values of 7.0 (A and B), 1.5 (C and D) and 0.5 (E and F).

thickness distributions

The film thickness distributions in Figure S7 are scaled with respect to the film thickness and are normalized such that the total area under each curve is equal to 1. The probability variable is used for the calculation of the percentage of film sub-thicknesses that are in a given range. In order to determine the percentage of film sub-thicknesses that are for example between the average value and two times the average value, the probability distribution function would be integrated between 1 and 2 and then multiplied by 100.

S5.4. Average FEM and deconstruction shape factor

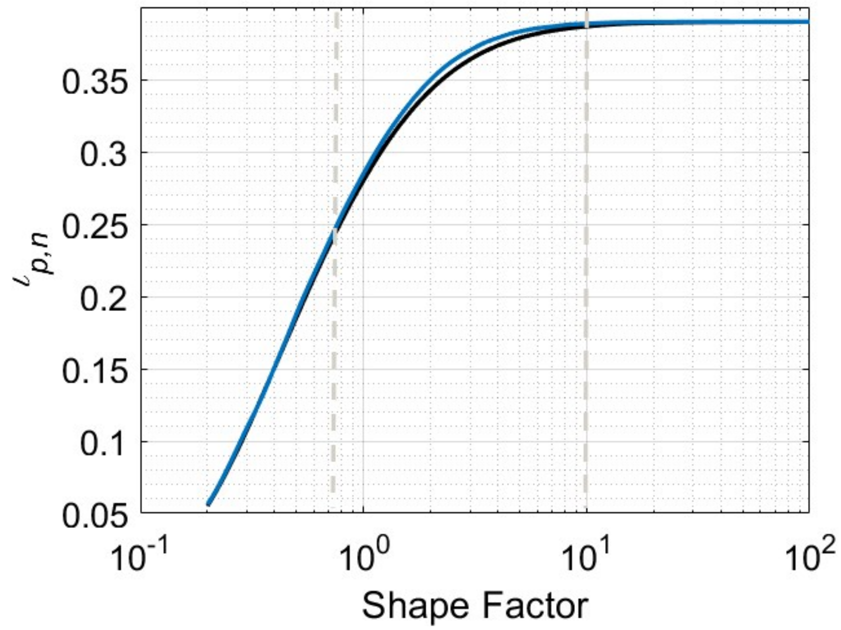

Figure S8. Overlay plot of the average FEM value (blue curve) and deconstruction (black curve) results at $w_{avg}^{1/2} = 2$, showing agreement between the two methods for shape factors less than 0.75 (left sidedashed grey line) or greater than 10 (right side dashed grey line).

correlation curve comparison

Based upon the agreement of the average FEM and deconstruction results for shape factors less than 0.75 (Figure S8), a normalized peak current overlay plot which allows for one to use normalized peak current values at values other than $w^{1/2} = 2$ for the estimation of the shape factor was prepared (Figure S9).

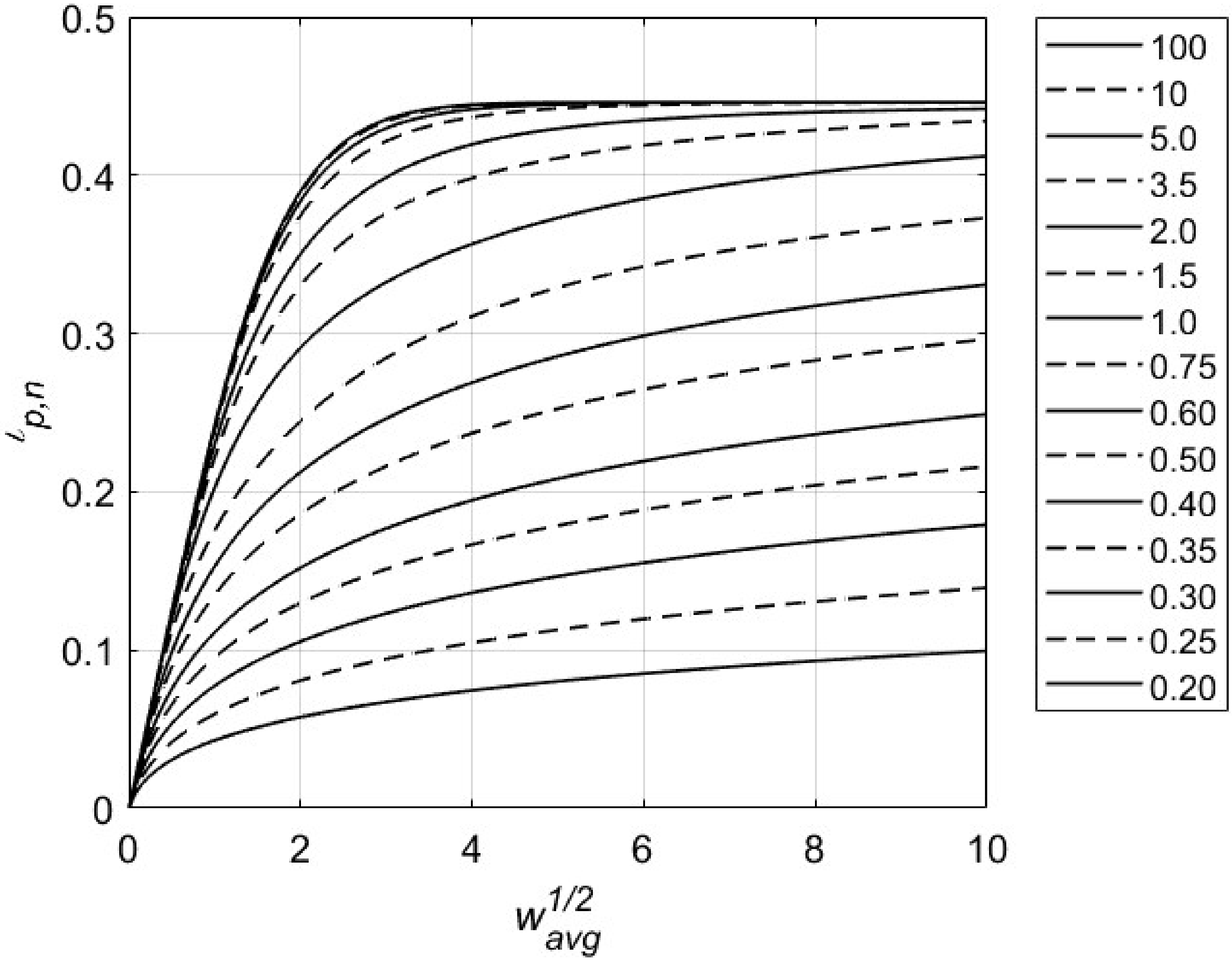

Figure S9. Normalized peak current plot for $w_{avg}^{1/2}$ between 0 and 10 and for shape factors ranging from 0.20 to 100 which takes hemisperical diffusion contributions into account. FEM was used for shape factors equal to 0.75 and greater, and deconstruction was used for shape factors below 0.75.

S5.5. Method resolution

The experimental current-time curves for the nine replicates used to establish the resolution of the method is shown in Figure S10. The corresponding peak currents, shape factors, and relative standard deviations are summarized in Table S1.

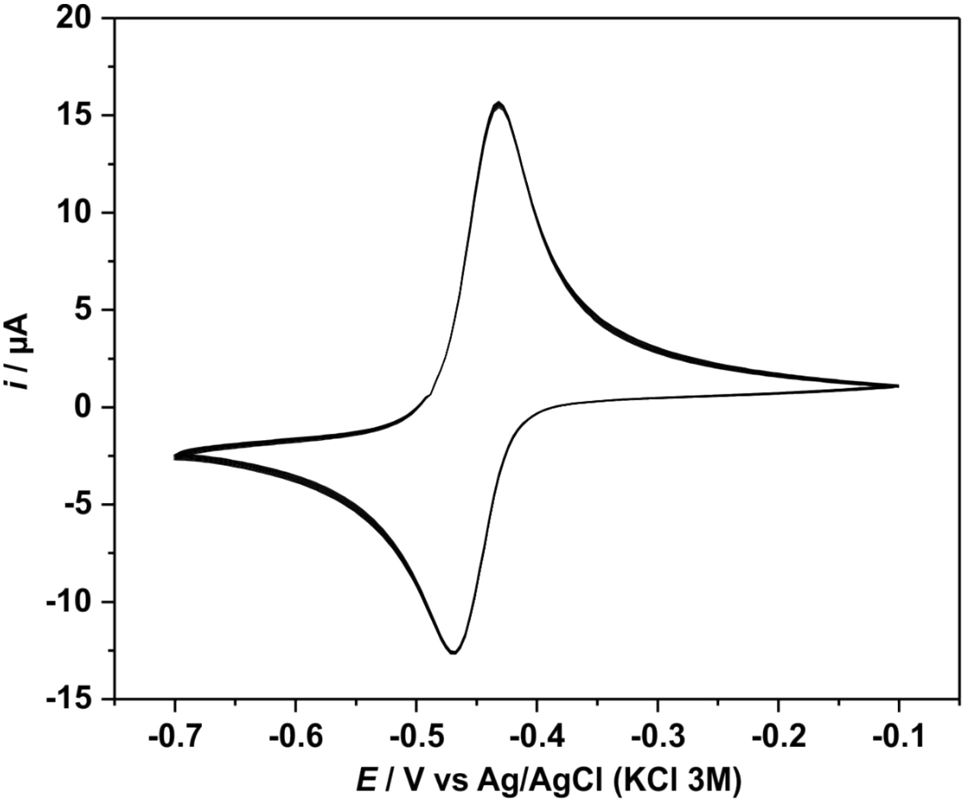

Figure S10: Cyclic voltammograms of viologen-modified dendrimer films. Successive 9 scans were performed under 100 % Ar at 298 K in phosphate buffer (0.1 M, pH 7) at a scan rate of 10 mV s^{-1}. Surface coverage of the dendrimer on the Au electrode was 0.3 mg cm^{-2}

Replicate	I_{pn}	Shape Factor	RSD
1	0.360	2.38	0.362
2	0.361	2.42	0.356
3	0.362	2.47	0.350
4	0.361	2.42	0.356
5	0.361	2.42	0.356
6	0.360	2.38	0.362
7	0.359	2.33	0.369
8	0.358	2.29	0.374
9	0.356	2.21	0.386
**Mean**	0.360	2.37	0.360
**Std Dev**	0.002	0.08	0.01

Table S1. Method resolution replicate data for the nine CVs shown in Figure S10, demonstrating that the method can be used to determine the shape factor with a resolution of 1%, based on the standard deviation of the replicates.

S6. Simplified procedure when slope and plateau are experimentally accessible

S6.1. Simplified non-dimensionalization

When the slope and plateau of the initial plot of $i_p/v^{1/2}$ vs $v^{1/2}$ are experimentally accessible, the corresponding dimensionless plot of $i_{p,n}$ vs $w_{avg}^{1/2}$ can be generated through the use of Equations S5-S8. Derivation of these equations took into consideration that in the slow scan rate region, the electrochemical response resembles that of an adsorbed species, and that in the plateau region, the electrochemical response resembles that of a semi-infinite diffusing species.

$$i_{p,n} = \left(\frac{i_p}{nFAC}\right)\left(\frac{RT}{nFvD}\right)^{1/2} = \left[\frac{(RT/nF)^{1/2}}{nFA\left(CD^{1/2}\right)}\right]\left(\frac{i_p}{v^{1/2}}\right) \tag{S5}$$

$$w_{avg}^{1/2} = d_{avg}\left[\frac{v}{D(nF/RT)}\right]^{1/2} = \left[\left(\frac{d_{avg}}{D^{1/2}}\right)\frac{1}{(RT/nF)^{1/2}}\right]v^{1/2} \tag{S6}$$

$$CD^{1/2} = \frac{(RT/nF)^{1/2}[Plateau]}{0.446nFA} \tag{S7}$$

$$\frac{d_{avg}}{D^{1/2}} = \frac{1.784\ (RT/nF)^{1/2}\ [Slope]}{[Plateau]} \tag{S8}$$

S6.2. Average thickness from transition scan rate

The intersection of the slope and plateau lines of the $i_{p,n}$ vs $w_{avg}^{1/2}$ plot is located at $w_{avg}^{1/2}$ = 1.6. This characteristic value can be used for calculation of the average film thickness through Equation S9 when one notes the transition scan rate v_T, which is the experimental scan rate that corresponds to the intersection of the slope and plateau lines of the $i_p/v^{1/2}$ vs $v^{1/2}$ plot.

$$d_{avg} = \frac{2.56(RT/nF)D}{\zeta\ v_T} \tag{S9}$$

S7. Accounting for interactions

Interactions within the hydrogel are quantified by the introduction of a general interaction parameter $\zeta = \Gamma_F/\Gamma_L$. The surface coverage which includes Frumkin interactions, Γ_F, is calculated using the experimental peak currents according to Equation S10 from slow scan-rate LSVs. The Langmuiran surface coverage, Γ_L, is calculated according Equation S11, where Q is the total charge obtained by integration of the chronoamperometry signal.

$$\Gamma_F = \frac{4\, \iota_p\, (RT/nF)}{n\, F\, A\, v} \tag{S10}$$

$$\Gamma_L = \frac{Q}{n\, F\, A} \tag{S11}$$

The film thickness is multiplied by ζ for the calculation of $w_{avg}^{1/2}$, according to Equation S12. If necessary, the calculated value of ζ can be tuned for optimal agreement between simulated and experimental data.

$$w_{avg}^{1/2} = (\,\zeta\, d_{avg})\left[\frac{v}{(RT/nF)\cdot D}\right]^{1/2} \tag{S12}$$

S8. Enlarged shape factor correlation plot with upper and lower limits

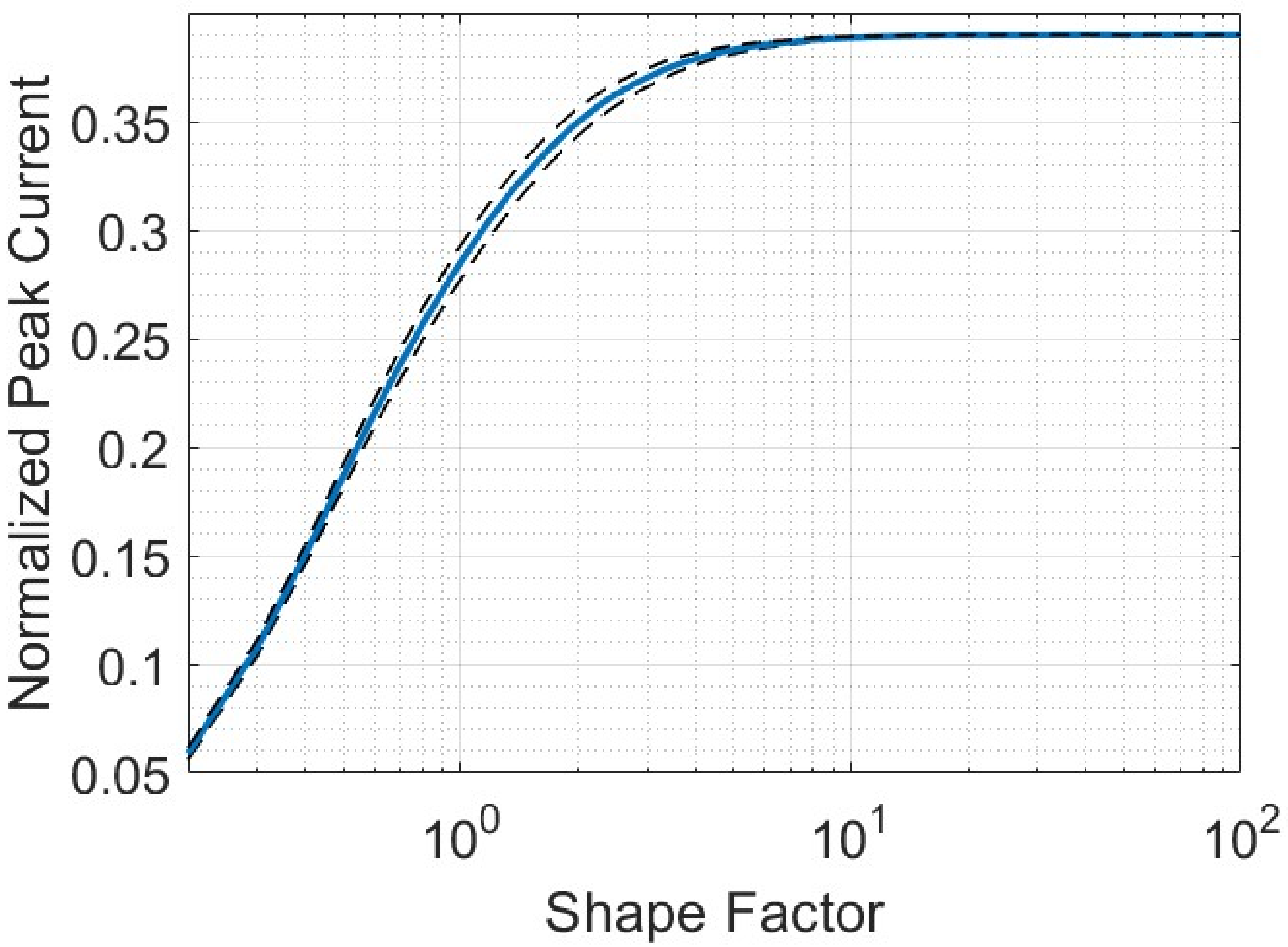

Figure S11. Correlation between normalized peak current at the characteristic value of $w_{avg}^{1/2} = 2$, and the corresponding Weibull Distribution shape factor. The average value (blue) has limits for the configuration with minimum hemispherical diffusion (dotted, to the left), and for the configuration with maximum hemispherical diffusion (dotted, to the right).

S9. Table of Symbols

Variable	Meaning	Example Units
δ	Diffusion layer thickness	cm
C_r	Scaled concentration of reduced redox species in the film	$Dimensionless$
$\bar{C}_r$	Concentration of redox species in the film (reduced form)	$mol \cdot cm^{-3}$
C	Concentration of the redox species within the film	$mol \cdot cm^{-3}$
g_{lh}	Length to height ratio of the redox film	*Dimensionless*
l	Film length (along x axis)	cm
d_{avg}	Average Film thickness	cm
x	Scaled x coordinate along the film width	*Dimensionless*
$\bar{x}$	x coordinate along the film width	cm
y	Scaled y coordinate along the film height	*Dimensioness*
$\bar{y}$	y coordinate along the film height	cm
t	Time	s
$\bar{t}$	Scaled time	*Dimensionless*
D	Diffusion coefficient for electron transfer within the film	$cm^2 \cdot s^{-1}$
v	Scan rate	$mV \cdot s^{-1}$
$\bar{v}$	Scaled scan rate	*Dimensionless*
v_T	Transition scan rate	$mV \cdot s^{-1}$
T	Temperature	K
A	Electrode surface area	cm^2
i_p	Peak current	A
$i_{p,n}$	Normalized peak current	*Dimensionless*
C	Total concentration of redox moiety in the film	$mol \cdot cm^{-3}$
R	Ideal gas constant	$mV \cdot \mu A \cdot mol^{-1} \cdot K^{-1}$
T	Temperature	K
n	Number of electrons transferred	*Dimensionless*
F	Faraday's constant	$\mu A \cdot s \cdot mol^{-1}$
E	Electrode potential	mV
E^0	Standard redox potential	mV
ζ	Interaction parameter	*Dimensionless*
Γ_F	Surface coverage (includes interactions)	$mol \cdot cm^{-2}$
Γ_L	Surface coverage (no interactions)	$mol \cdot cm^{-2}$
Q	Total charge	C

Table S2. Table of symbols

S10. References

(1) Nečas, D.; Klapetek, P. Gwyddion: an open-source software for SPM data analysis. *Open Physics* 2012, **10**, 99.

(2) Rinne, H. The Weibull Distribution: A handbook; CRC Press: Boca Raton, 2009.

(3) Gadelmawla, E. S.; Koura, M. M.; Maksoud, T.M.A.; Elewa, I. M.; Soliman, H. H. Roughness Parameters. *J. Mater. Proc. Tech.* 2002, **123**, 133.

(4) Aoki, K.; Tokuda, K.; Matsuda, H. Theory of Linear Sweep Voltammetry with Finite Diffusion Space. *J. Electroanal. Chem.* 1983, **146**, 417.
